# Enantioselective Addition of Allyltin Reagents to Amino Aldehydes Catalyzed with Bis(oxazolinyl)phenylrhodium(III) Aqua Complexes

**DOI:** 10.3390/molecules16075387

**Published:** 2011-06-27

**Authors:** Yukihiro Motoyama, Takatoshi Sakakura, Toshihide Takemoto, Kayoko Shimozono, Katsuyuki Aoki, Hisao Nishiyama

**Affiliations:** 1 Institute for Materials Chemistry and Engineering, Kyushu University, 6-1 Kasuga-koen, Kasuga, Fukuoka 816-8580, Japan; 2 School of Materials Science, Toyohashi University of Technology, Tempaku-cho, Toyohashi, Aichi 441-8580, Japan; 3 Department of Applied Chemistry, Graduate School of Engineering, Nagoya University, Chikusa, Nagoya 464-8603, Japan

**Keywords:** allylation, amino aldehydes, Lewis acids, pincer ligands, rhodium

## Abstract

Bis(oxazolinyl)phenylrhodium(III) aqua complexes, (Phebox)RhX_2_(H_2_O) [X = Cl, Br], were found to be efficient Lewis acid catalysts for the enantioselective addition of allyl- and methallyltributyltin reagents to amino aldehydes. The reactions proceed smoothly in the presence of 5–10 mol % of (Phebox)RhX_2_(H_2_O) complex at ambient temperature to give the corresponding amino alcohols with modest to good enantioselectivity (up to 94% ee).

## 1. Introduction

**Figure 1:**
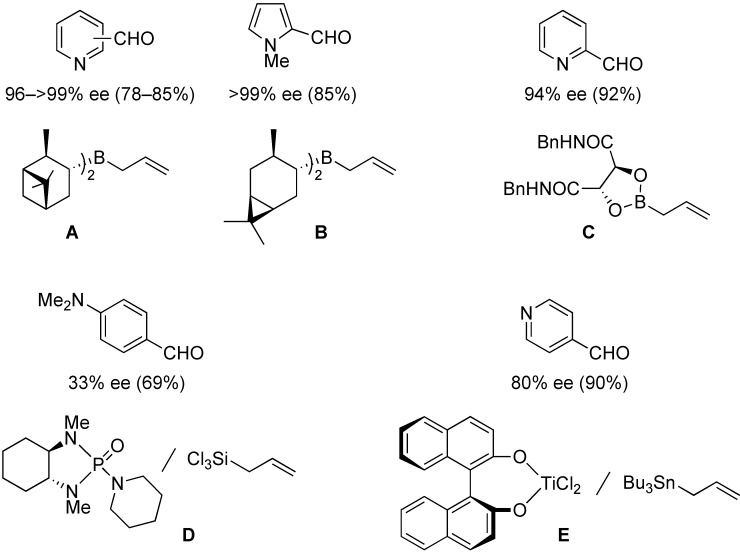
Chiral allylmetal reagents for the asymmetric allylation of amino aldehydes.

The development of enantioselective synthesis of chiral homoallylic alcohols containing amino-functional groups is of great importance to synthetic organic and medicinal chemistry. Despite much effort directed at enantioselective allylation of aldehydes [1,2,3], there are only a few systems for enantioselective allylation of amino aldehydes as substrates because the high coordination ability of amino groups to the metal species often leads to deactivation of chiral allylmetals or catalysts. Therefore, most of these reactions need a stoichiometric amount of chiral sources. For examples, Brown [4] and Chen [5] reported the utility of allylboron reagents (Figure 1, **A**–**C**) for the reaction with pyridinecarboxaldehydes and 1-methyl-2-pyrrolecarboxaldehyde [4,5]. Denmark and co-workers developed a new reaction system for the allylation of aldehydes, but the enantioselectivity of the reaction with 4-dimethylaminobenzaldehyde was not so high (Figure 1, **D**) [6]. The other one is a catalytic reaction using 20 mol % of BINOL-derived chiral titanium complex/allyltributyltin *via* transmetalation mechanism reported by Umani-Ronchi (Figure 1, **E**) [7]. 

**Scheme 1:**
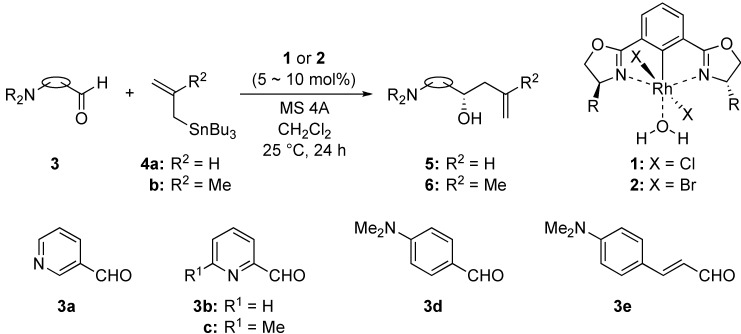
Enantioselective addition of allyltin reagents to amino aldehydes catalyzed with (Phebox)RhX_2_(H_2_O) complexes (X = Cl, Br).

We have previously developed a meridional tridentate ligand, 2,6-bis(oxazolinyl)phenyl derivative (abbreviated to Phebox) as a chiral N–C–N pincer type ligand with one central covalent bond to a metal [8,9,10,11,12,13,14], and have demonstrated that rhodium(III) aqua complexes bearing the Phebox ligand, (Phebox)RhX_2_(H_2_O) [1: X = Cl, 2: X = Br], acted as recoverable chiral Lewis acid catalysts for the enantioselective addition of allylic tributyltin reagents to aldehydes [15,16,17] and the asymmetric hetero Diels-Alder reactions of Danishefsky’s dienes and glyoxylates [18]. During the course of our studies on the Phebox-Rh(III) system as a chiral transition metal Lewis acid, we have found that tertiary amines such as *N,N*-diisopropylethylamine or triethylamine cannot bind to the rhodium atom [19]. This discovery encouraged us to use these air-stable and water-tolerant complexes **1** and **2** for the allylation of amino aldehydes as substrates. We wish to report herein the Lewis acid-catalyzed enantioselective addition of allyl- and methallyltributyltin reagents to aldehydes containing amino-functional groups (Scheme 1). 

## 2. Results and Discussion

### 2.1. NMR Studies, Isolation, and X-ray Analysis of Phebox-Rh(III)–Amino Aldehyde Complexes

**Scheme 2:**
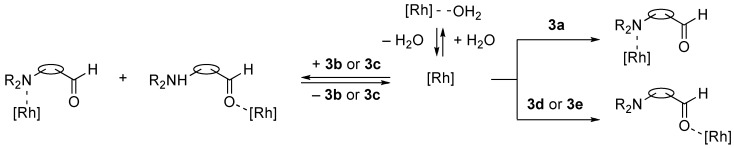
Complexation between (Phebox)RhCl_2_ fragment ([Rh]) and amino aldehydes.

**Figure 2:**
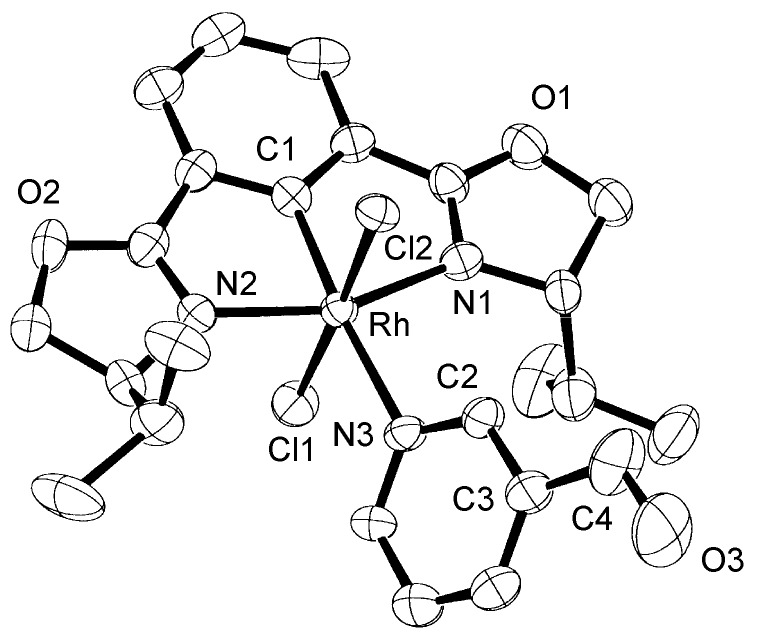
Molecular structure of (*i*-Pr-Phebox)RhCl_2_(κ-**3a**): there are two independent molecules and one H_2_O in the unit cell.

First, we checked the complexation between Phebox-Rh(III) complex *i*-Pr-**1** and amino aldehydes **3a**–**e** by ^1^H and ^13^C-NMR. Selected ^1^H and ^13^C-NMR data in CDCl_3_are listed in Table 1. Although rigid complexation was not clearly observed between *i*-Pr-**1** and 6-methyl-2-pyridinecarboxaldehyde (**3c**) (Entry 3), ^1^H- and ^13^C-NMR spectra of the other cases showed formation of new complexes. From the NMR spectra of a mixture of *i*-Pr-**1** and **3a**, the pyridine’s nitrogen atom exclusively forms σ-complexes with the (*i*-Pr-Phebox)RhCl_2_ fragment; the signals of the protons for 2- and 6-positions of the pyridine ring (H_2_ and H_6_) appeared at lower field than those of the uncomplexed (free) **3a** (from δ 9.08 to 10.30 ppm for H_2_, and from δ 8.85 to 10.08 ppm for H_6_, respectively) (Entry 1). This amine complex was stable enough to be purified by silica gel chromatography and was eventually characterized by a single-crystal X-ray diffraction (Figure 2, Table 2). In the case of the reaction of *i*-Pr-**1** and **3b**, H_6_ and the formyl proton (H_f_) both appeared as broad signals at lower field (δ 9.11 for H_6_ and 10.34 ppm for H_f_) than those of free **3b**(δ 8.77 for H_6_ and 10.07 ppm for H_f_) (Entry 2). These results indicate that the coordination of **3b** to the (*i*-Pr-Phebox)RhCl_2_ fragment is an equilibrium between the pyridinic nitrogen and carbonyl oxygen. In contrast to the pyridinecarboxaldehydes, solutions of 4-dimethylaminobenzaldehyde (**3d**) and 4-dimethylaminocinnamaldehyde (**3e**) in the presence of *i*-Pr-**1** showed rigid formation of C=O/σ type aldehyde complexes. For example, the signals assignable to the dimethylamino group were not changed, but the signals of the formyl proton (H_f_) and carbon (C_f_) of coordinated **3d** appeared at lower field than the uncomplexed (free) **3d** (from δ 9.74 to 9.92 ppm for H_f_, and from δ 190.4 to 207.2 ppm for C_f_, respectively) (Entry 4). Similar lower field shifts of H_f_ and C_f_ along with the olefinic protons H_α_ and H_β_ (H_α_ = α-proton, H_β_ = β-proton) were also observed for the mixture of *i*-Pr-**1** and **3e** in ^1^H and ^13^C NMR (ΔH_α_ = +0.27 ppm, ΔH_β_ = +0.14 ppm, ΔH_f_ = +0.77 ppm, ΔC_f_ = +2.9 ppm, respectively) (Entry 5). It is widely known that chemical shifts of vinylic protons (H_α_ and H_β_), formyl proton (H_f_) and carbon (C_f_) of enals bound to Lewis acids by the carbonyl oxygen appear at lower field than those of free enals [20,21,22]. These lower-field shifts of H_α_, H_β_, H_f_ and C_f_ are also observed in the reaction of *i*-Pr-**1** and (*E*)-cinnamaldehyde [16]. The above NMR and X-ray studies thus indicated that the (Phebox)RhCl_2_ fragment, generated by releasing H_2_O from (Phebox)RhCl_2_(H_2_O), captures amino aldehydes **3b–e** at the carbonyl oxygen to form aldehyde complexes (Scheme 2).

**Table 1:** Selected spectroscopic data for free amino aldehydes **3** and mixtures of *i*-Pr-**1** and **3**.

Entry		δ (ppm) ^a^		Δ (ppm) ^b^
		3	*i*-Pr-1 and 3	
1	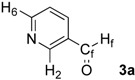	H_2_: 9.08	H_2_: 10.30	+1.22
		H_6_: 8.85	H_6_: 10.08	+1.23
		H_f_: 10.12	H_f_: 10.27	+0.15
		C_f_: 190.8	C_f_: 189.8	–1.0
2	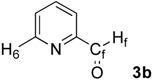	H_6_: 8.77	H_6_: 9.11 (br)	+0.34
		H_f_: 10.07	H_f_: 10.34 (br)	+0.27
3	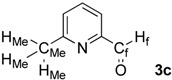	H_Me_: 2.66	H_Me_: 2.66	0.00
		H_f_: 10.04	H_f_: 10.06	+0.02
		C_Me_: 24.5	C_Me_: 24.5	0.0
		C_f_: 193.1	C_f_: 194.1	+1.0
4	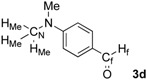	H_Me_: 3.09	H_Me_: 3.11	+0.02
		H_f_: 9.74	H_f_: 9.92	+0.18
		C_N_: 40.2	C_N_: 40.2	0.0
		C_f_: 190.4	C_f_: 207.2	+16.8
5	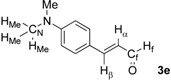	H_α_: 6.54	H_α_: 6.81	+0.27
		H_β_: 7.38	H_β_: 7.52	+0.14
		H_Me_: 3.05	H_Me_: 3.07	+0.02
		H_f_: 9.09	H_f_: 9.86	+0.77
		C_N_: 40.2	C_N_: 40.2	0.0
		C_f_: 193.8	C_f_: 196.7	+2.9

^a^ Observed at 400 MHz for ^1^H-NMR and 100 MHz for ^13^C-NMR in CDCl_3_ at ambient temperature; ^b^ Calculated by δ (*i*-Pr-**1** and **3**) – δ (**3**).

**Table 2:** Selected bond distances (Å) and angles (deg) for (*i*-Pr-Phebox)RhCl_2_(κ-**3a**).^a^

Rh–C1	1.93(1) [1.89(1)]	Rh–N1	2.05(1) [2.06(1)]
Rh–Cl1	2.340(4) [2.334(4)]	Rh–N2	2.05(1) [2.09(1)]
Rh–Cl2	2.334(4) [2.351(4)]	Rh–N3	2.21(1) [2.27(1)]
		C4–O3	1.25(3) [1.27(4)]
C1–Rh–N3	175.2(6) [178.0(5)]	N1–Rh–N3–C2	54(1) [90(1)]
Cl1–Rh–Cl2	178.0(2) [177.2(2)]	O3–C4–C3–C2	−175(2) [19(3)]
N1–Rh–N2	158.4(5) [157.6(5)]		

^a^ Bond distances and angles of the second molecule are given in brackets.

### 2.2. Phebox-Rh(III)-Catalyzed Enantioselective Addition of Allyltributyltin to Amino Aldehydes

**Scheme 3:**
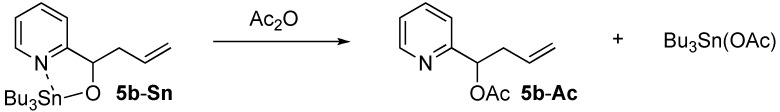
Conversion of **5b**-**Sn** to **5b**-**Ac** by treatment with Ac_2_O.

We also examined the Phebox-Rh(III)-catalyzed reaction of amino aldehydes and allyltributyltin. Allyltributyltin (**4a**) was added to a suspension of 4Å molecular sieves (MS 4A), amino aldehydes **3b**–**e** and 5–10 mol % of (*S*,*S*)-(Phebox)RhX_2_(H_2_O) complexes (**1 **or **2**) in dichloromethane at 25 °C for 24 h. These results are summarized in Table 3. First, the reaction of pyridine-2-carboxaldehyde (**3b**) proceeded smoothly, but the isolated yield of the allylated product **5b** was only 14% after purification of the crude material by silica gel chromatography (Entry 1). This result indicates that the alkoxystananne **5b**-**Sn**formed in the reaction mixture is stable and hardly hydrolyzed under the usual workup process (see Experimental section). Consequently, we adopted a new procedure for conversion of **5b**-**Sn** to the acetate derivative **5b**-**Ac** by treatment with acetic anhydride (Scheme 3). In this manner, **5b**-**Ac** was obtained in good to high yields and with moderate enantioselectivity (Entries 2, 3, 5, and 6). The absolute configuration of **5b**-**Ac** obtained by (*S,S*)-Phebox-derived Rh(III) complexes was determined to be *S* by comparison of the optical rotation value with literature data [23]. In the case of the reaction using (*S,S*)-Ph-**1**, however, *ca*. 50% of **3b** was recovered after silica gel chromatography and (*R*)-**5b**-**Ac** was formed as a major enantiomer (Entry 4). Finally, an enantioselectivity of up to 59% ee was achieved using 10 mol % of the *i*-Pr- and Me-Phebox-derived dibromide complexes (Entries 5 and 6). In the cases of the other aldehydes **3c**–**e**, the products were obtained as a homoallylic alcohol in good to high yields. The reactions of 6-methyl-2-pyridinecarboxaldehyde (**3c**) and 4-dimethylaminocinnamaldehyde (**3e**) afforded the corresponding amino alcohols **5c** and **5e** with good enantioselectivity by using the dibromide complexes (**5c:** 84% ee with Ph-**2**, and **5e:** 88% ee with Bn-**2**, respectively) (entries 9 and 15). In sharp contrast, the dichloride complexes showed higher enantioselectivity (84% ee for Me-**1**, and 81% ee for *s*-Bu-**1**, respectively) than the parent dibromide one (72% ee for Me-**2**) in the allylation reaction of 4-dimethylamino- benzaldehyde (**3d**) (Entries 10–12). We also examined the reaction of 3-pyridinecarboxaldehyde (**3a**), however, no allylated product was obtained and the amine complex, (*i*-Pr-Phebox)RhCl_2_(κ-**3a**), free **3a**, and allyltributyltin (**4a**) were detected by ^1^H-NMR of the crude material.

**Table 3:** Enantioselective addition of allyltributyltin **4a** to amino aldehydes**3b**–**e**.^a^

Entry	Aldehyde	Catalyst	Product	% Yield	% ee^b^ (config.) ^c^
1	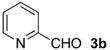	*i*-Pr-**1**	**5b**	14	42 (*S*)
2 ^d^		*i*-Pr-**1**	**5b-Ac**	99	53 (*S*)
3 ^d^		Me- **1**	**5b-Ac**	99	56 (*S*)
4 ^d^		Ph- **1**	**5b-Ac**	45	21 (*R*)
5 ^d^		*i*-Pr-**2**	**5b-Ac**	81	59 (*S*)
6 ^d^		Me- **2**	**5b-Ac**	85	59 (*S*)
7	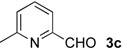	*i*-Pr-**1**	**5c**	94	69 (*S*)^e^
8		Ph- **1**	**5c**	89	75 (*S*)^e^
9		Ph- **2**	**5c**	97	84 (*S*)^e^
10	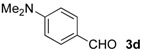	Me- **1**	**5d**	80	84 (*S*)
11		*s*-Bu-**1**	**5d**	67	81 (*S*)
12		Me- **2**	**5d**	42	72 (*S*)
13	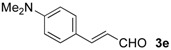	Bn- **1**	**5e**	80	81 (*S*)^e^
14		*s*-Bu-**1**	**5e**	61	80 (*S*)^e^
15		Bn- **2**	**5e**	44	88 (*S*)^e^

^a^ All reactions were carried out using 0.5 mmol of **3**, 0.75 mmol of **4**, and 0.025 mmol (5 mol %) of **1** or 0.05 mmol (10 mol %) of **2** in 2 mL of dichloromethane in the presence of MS 4A (250 mg) at 25 °C for 24 h; ^b^ Determined by chiral HPLC analysis using Daicel CHIRALCEL OD; ^c^ Assignment by comparison of the sign of optically rotation with reported value; ^d^ 0.6 mmol of acetic anhydride was added; ^e^ Assignment by analogy.

### 2.3. Phebox-Rh(III)-Catalyzed Enantioselective Addition of Methallyltributyltin to Amino Aldehydes

Table 4 summarizes the results obtained for the methallylation of amino aldehydes **3b**–**e** catalyzed with Phebox-Rh(III) complexes in dichloromethane in the presence of MS 4A at 25 °C for 24 h. In the reactions of pyridinecarboxaldehydes **3b** and **3c**, the enantiomeric excesses of the methallylated products **6b**-**Ac** and **6c** were moderate (51% ee for **6b**-**Ac**, and 45% ee for **6c**, respectively) (Entries 1–10). Similar to the reaction of pyridine-2-carboxaldehyde **3b** and allyltributyltin (Table 2, Entry 4), (*S,S*)-Ph-**1** afforded the opposite (*R*)-**6b**-**Ac** as a major enantiomer (Entry 3). Compared to the reactions with pyridinecarboxaldehydes **3b** and **3c**, the (*S*,*S*)-Phebox-Rh-catalyzed reactions of 4-dimethyl-aminobenzaldehyde (**3d**) and 4-dimethylaminocinnamaldehyde (**3e**) with methallyltributyltin (**4b**) afforded the corresponding (*S*)-products with good to high enantioselectivity (90% ee for **6d** and 94% ee for **6e**, respectively) (Entries 11–17). Incidentally, the Phebox-Rh(III) aqua complexes **1** and **2** can be recovered almost quantitatively from the reaction media by silica gel column chromatography.

**Table 4:** Enantioselective addition of methallyltributyltin **4b** to amino aldehydes **3b**–**e**.^a^

Entry	Aldehyde	Catalyst	Product	% Yield	% ee ^b^ (config.) ^c^
1 ^d^	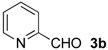	Bn- **1**	**6b-Ac**	79	15 (*S*)
2 ^d^		Me- **1**	**6b-Ac**	76	41 (*S*)
3 ^d^		Ph- **1**	**6b-Ac**	18	24 (*R*)
4 ^d^		*s*-Bu-**1**	**6b-Ac**	52	<2 (–)
5 ^d^		Me- **2**	**6b-Ac**	48	45 (*S*)
6 ^d^		*s*-Bu-**2**	**6b-Ac**	22	51 (*S*)
7	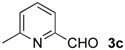	Me- **1**	**6c**	60	45 (*S*)^e^
8		*s*-Bu-**1**	**6c**	36	11 (*S*)^e^
9		Me- **2**	**6c**	21	10 (*S*)^e^
10		*s*-Bu-**2**	**6c**	26	26 (*S*)^e^
11	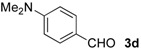	*i*-Pr-**1**	**6d**	84	85 (*S*)^e^
12		Bn- **1**	**6d**	79	90 (*S*)^e^
13		*s*-Bu-**1**	**6d**	68	87 (*S*)^e^
14		Bn- **2**	**6d**	52	63 (*S*)^e^
15	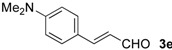	*i*-Pr-**1**	**6e**	52	80 (*S*)^e^
16		*s*-Bu-**1**	**6e**	74	84 (*S*)^e^
17		*s*-Bu-**2**	**6e**	20	94 (*S*)^e^

^a^ All reactions were carried out using 0.5 mmol of **3**, 0.75 mmol of **4**, and 0.025 mmol (5 mol %) of **1** or 0.05 mmol (10 mol %) of **2** in 2 mL of dichloromethane in the presence of MS 4A (250 mg) at 25 °C for 24 h; ^b^ Determined by chiral HPLC analysis using Daicel CHIRALCEL OD; ^c^ Assignment by comparison of the sign of optically rotation with reported value; ^d^ 0.6 mmol of acetic anhydride was added; ^e^ Assignment by analogy.

## 3. Experimental

### 3.1. General

Anhydrous dichloromethane was purchased from Kanto Chemical Co. Carbon tetrabromide, all aldehydes and allyltributyltin were purchased from Tokyo Chemical Industry Co., Ltd. ^1^H and ^13^C NMR spectra were measured on a VARIAN Inova-400 (400 MHz) spectrometer. ^1^H-NMR (400 MHz) chemical shifts were described in parts per million downfield from tetramethylsilane used as an internal standard (δ = 0) in CDCl_3_, unless otherwise noted. ^13^C-NMR (100 MHz) chemical shifts were expressed in parts per million with reference to the residual solvent peak as an internal standard (δ = 77.1 for CDCl_3_), unless otherwise noted. IR spectra were measured on a JASCO FT/IR-230 spectrometer. Melting points were measured on a Yanaco MP-J3 apparatus. Elemental analyses were measured on a Yanaco CHN CORDER MT-6 instrument. High performance liquid chromatography (HPLC) analyses were performed with a JASCO PU-980 HPLC pump, UV-975 and 980 UV/VIS detector, and CO-966 column thermostat (at 25 °C) using a Daicel CHIRALCEL OD column. Optical rotations were measured on a JASCO DIP-140 polarimeter. Column chromatography was performed with silica gel (Merck, Art. No. 7734). Analytical thin-layer chromatography (TLC) was performed on glass plates and aluminum sheets pre-coated with silica gel (Merck, Kieselgel 60 F-254, layer thicknesses 0.25 and 0.2 mm, respectively). Visualization was accomplished by UV light (254 nm), anisaldehyde, and phosphomolybdic acid. All reactions were carried out under a nitrogen or argon atmosphere. (Phebox)SnMe_3_, (Phebox)RhCl_2_(H_2_O) **1** and (Bn-Phebox)RhBr_2_(H_2_O) (Bn-**2**) were prepared by our method [16,18,24]. [(*c*-octene)_2_RhCl]_2_[25] and methallyltributyltin [26] were prepared by the literature methods. 

### 3.2. General Procedure for the Synthesis of (Phebox)RhBr_2_(H_2_O) Complexes

To a stirred solution of (R-Phebox)SnMe_3_ and [(*c*-octene)_2_RhCl]_2_ in dichloromethane was added tetrabromomethane [*ca*. 8 equivalents with respect to (R-Phebox)SnMe_3_] at ambient temperature for 24 h. Then the reaction mixture was concentrated under reduced pressure. Purification of the residue by silica gel chromatography gave (R-Phebox)RhBr_2_(H_2_O) (R-**2**). 

*(i-Pr-Phebox)RhBr_2_(H_2_O)* (*i*-Pr-**2**). 51% yield. Pale yellow solid. mp. 167 °C (decomp); IR (KBr) ν 3474, 2959, 1622, 1485, 1391, 961, 745 cm^−1^; ^1^H-NMR (CDCl_3_) δ 0.94 (d, *J* = 6.5 Hz, 6H), 0.96 (d, *J* = 7.0 Hz, 6H), 2.42 (qqd, *J* = 7.0, 6.5, 2.5 Hz, 2H), 3.43 (bs, 2H), 4.30 (td, *J* = 8.3, 2.5 Hz, 2H), 4.71 (d, *J* = 8.3 Hz, 4H), 7.25 (t, *J* = 7.6 Hz, 1H), 7.58 (d, *J* = 7.6 Hz, 2H); ^13^C-NMR (CDCl_3_) δ 15.4, 19.6, 29.1, 67.5, 71.3, 123.1, 128.1, 131.5, 170.9 (d, *J*_Rh-C_ = 4.1 Hz), 178.8 (d, *J*_Rh-C_ = 24.2 Hz); Anal. C_18_H_25_N_2_O_3_Br_2_Rh: Found C 37.31, H 4.33, N 4.74%; Calcd C 37.27, H 4.34, N 4.83%.

*(Me-Phebox)RhBr_2_(H_2_O)* (Me-**2**). 58% yield. Orange solid; mp. >300 °C (decomp); IR (KBr) ν 3397, 3009, 2822, 1617, 1485, 1397, 1148, 958, 739 cm^−1^; ^1^H-NMR (CD_3_OD) δ 1.50 (d, *J* = 6.7 Hz, 6H), 3.32 (bs, 2H), 4.34 (ddq, *J* = 8.8, 7.7, 6.7 Hz, 2H), 4.45 (dd, *J* = 8.5, 7.7 Hz, 2H), 5.02 (dd, *J* = 8.8, 8.5 Hz, 2H), 7.24 (t, *J* = 7.7 Hz, 1H), 7.58 (d, *J* = 7.7 Hz, 2H); ^13^C-NMR (CD_3_OD) δ 19.3, 58.6, 77.7, 122.3, 126.9, 132.4, 171.1 (d, *J*_Rh-C_ = 3.7 Hz), 182.2 (d, *J*_Rh-C_ = 20.0 Hz); Anal. C_14_H_17_N_2_O_3_Br_2_Rh: Found C 32.04, H 3.39, N 5.27%; Calcd C 32.09, H 3.27, N 5.35%.

*(Ph-Phebox)RhBr_2_(H_2_O)* (Ph-**2**). 37% yield. Pale yellow solid. mp. 189 °C (decomp); IR (KBr) ν 3452, 2980, 2825, 1613, 1485, 1326, 1149, 968 cm^−1^; ^1^H-NMR (CDCl_3_) δ 1.79 (bs, 2H), 4.58 (dd, *J* = 10.4, 8.6 Hz, 2H), 5.18 (dd, *J* = 10.2, 8.6 Hz, 2H), 5.31 (dd, *J* = 10.4, 10.2 Hz, 2H), 7.35-7.46 (m, 7H), 7.46-7.74 (m, 4H), 7.72 (d, *J* = 7.7 Hz, 2H); ^13^C-NMR (CDCl_3_) δ 31.7, 67.1, 76.8, 123.3, 128.5, 128.8, 128.9, 131.4, 137.4, 172.5 (d, *J*_Rh-C_ = 4.2 Hz), 180.4 (d, *J*_Rh-C_ = 21.1 Hz); Anal. C_24_H_21_N_2_O_3_Br_2_Rh: Found C 44.48, H 3.19, N 4.30%; Calcd C 44.47, H 3.27, N 4.32%.

*(s-Bu-Phebox)RhBr_2_(H_2_O)* (s-Bu-**2**). 21% yield. Pale yellow solid. mp. 119 °C (decomp); IR (KBr) ν 3448, 2968, 2822, 1617, 1484, 1394, 1145, 963 cm^−1^; ^1^H-NMR (CDCl_3_) δ 0.96 (d, *J* = 6.8 Hz, 6H), 1.00 (t, *J* = 7.3 Hz, 6H), 1.24 (m, 2H), 1.39 (m, 2H), 2.17 (m, 2H), 3.43 (bs, 2H), 4.34 (ddd, *J* = 9.9, 6.7, 3.2 Hz, 2H), 4.69 (dd, *J* = 8.8, 6.7 Hz, 2H), 4.74 (dd, *J* = 9.9, 8.8 Hz, 2H), 7.26 (t, *J* = 7.7 Hz, 1H), 7.59 (d, *J* = 7.7 Hz, 2H); ^3^C-NMR (CDCl_3_) δ 12.0, 12.8, 35.8, 66.4, 71.4, 77.4, 123.4, 128.2, 131.4, 170.7 (d, *J*_Rh-C_ = 4.2 Hz), 176.9 (d, *J*_Rh-C_ = 24.5 Hz); Anal. C_20_H_29_N_2_O_3_Br_2_Rh: Found C 39.51, H 4.73, N 4.63%; Calcd C 39.50, H 4.81, N 4.61%.

### 3.3. General Procedure for the Catalytic Enantioselective Addition of Allyl- or Methallyltributyltin to Aldehydes Catalyzed with (Phebox)RhX_2_(H_2_O) Complexes

To a suspension of MS 4A (250 mg) in dichloromethane (2 mL) was added (Phebox)RhX_2_(H_2_O) complex (0.025-0.05 mmol, 5-10 mol %), amino aldehyde (0.5 mmol) and allyl- or methallyltributyltin (0.75 mmol) at 25 °C. After it was stirred for 24 h at that temperature, the reaction mixture was concentrated under reduced pressure. Purification of the residue by silica gel chromatography gave homoallylic alcohol: the enantioselectivity was determined by chiral HPLC analysis.

*1-(2-Pyridyl)-3-buten-1-ol* (**5b**) [4,5]. [α]_D_^20^ −27.1° (c 1.22, CHCl_3_) for 42% ee: lit. 4 [α]_D_^23^ −32.5° (c 3.5, EtOH) for ≥99% ee, 1S; IR (neat) ν 3267, 2922, 1733, 1699, 164, 1562, 1474, 1066, 702 cm^−1^; ^1^H- NMR (CDCl_3_) δ 2.48 (ddddd, *J* = 14.5, 7.3, 6.9, 1.3, 1.1 Hz, 1H), 2.63 (ddddd, *J* = 14.5, 6.9, 4.7, 1.4, 1.2 Hz, 1H), 4.07 (bs, 1H), 4.81 (bs, 1H), 5.09 (dddd, *J* = 10.1, 2.0, 1.2, 1.1 Hz, 1H), 5.11 (dddd, *J* = 17.2, 2.0, 1.4, 1.3 Hz, 1H), 5.83 (dddd, *J* = 17.2, 10.1, 7.3, 6.9 Hz, 1H), 7.20 (ddd, *J* = 7.5, 4.9, 1.2 Hz, 1H), 7.28 (ddd, *J* = 7.8, 1.2, 1.0 Hz, 1H), 7.68 (ddd, *J* = 7.8, 7.5, 1.5 Hz, 1H), 8.54 (ddd, *J* = 4.9, 1.5, 1.0 Hz, 1H); ^13^C-NMR (CDCl_3_) δ 43.0, 72.3, 118.1, 120.5, 122.4, 134.2, 136.7, 148.4, 161.4. Enantiomeric excess was determined by after conversion to the corresponding acetate **5b**-Ac.

*1-Acetoxy-1-(2-pyridyl)-3-butene* (**5b**-**Ac**) [23]. IR (neat) ν 1738, 1592, 1372, 1235, 1047, 921 cm^−1^; ^1^H-NMR (CDCl_3_) δ 2.13 (s, 3H), 2.69 (ddddd, *J* = 14.3, 7.5, 6.9, 1.3, 1.1 Hz, 1H), 2.76 (ddddd, *J* = 14.3, 7.1, 5.8, 1.4, 1.2 Hz, 1H), 5.04 (dddd, *J* = 10.2, 1.9, 1.2, 1.1 Hz, 1H), 5.08 (dddd, *J* = 17.2, 1.9, 1.4, 1.3 Hz, 1H), 5.74 (dddd, *J* = 17.2, 10.2, 7.1, 6.9 Hz, 1H), 5.86 (dd, *J* = 7.5, 5.8 Hz, 1H), 7.20 (ddd, *J* = 7.6, 4.8, 1.3 Hz, 1H), 7.30 (ddd, *J* = 7.9, 1.3, 0.9 Hz, 1H), 7.67 (ddd, *J* = 7.9, 7.6, 1.8 Hz, 1H), 8.60 (ddd, *J* = 4.8, 1.8, 0.9 Hz, 1H); ^13^C-NMR (CDCl_3_) δ 21.2, 39.2, 75.7, 118.2, 121.3, 122.8, 133.2, 136.7, 149.5, 158.9, 170.4; [α]_D_^20^ −40.8° (c 1.10, CHCl_3_) for 59% ee: lit. [α]_D_^25^ +75° (c 2.01, CHCl_3_) for 92% ee, 1*R* [23]; Daicel CHIRALCEL OD, UV Detector 254 nm, hexane/i-PrOH = 9:1, flow rate 0.5 mL/min. t_R_ = 10.2 min (*R*), 12.8 min (*S*). 

*1-(6-Methyl-2-pyridyl)-3-buten-1-ol* (**5c**). IR (neat) ν 3417, 2907, 1642, 1594, 1459, 1066, 799 cm^−1^; ^1^H-NMR (CDCl_3_) δ 2.45 (dtt, *J* = 14.2, 7.0, 1.1 Hz, 1H), 2.55 (s, 3H), 2.61 (dddt, *J* = 14.2, 6.9, 4.8, 1.1 Hz, 1H), 4.40 (d, *J* = 4.8 Hz, 1H), 4.76 (dt, *J* = 7.0, 4.8 Hz, 1H), 5.01 (ddt, *J* = 10.1, 2.0, 1.2 Hz, 1H), 5.12 (ddt, *J* = 17.1, 2.0, 1.5 Hz, 1H), 5.85 (ddt, *J* = 17.1, 10.1, 7.0 Hz, 1H), 7.04 (d, *J* = 7.7 Hz, 1H), 7.06 (d, *J* = 7.7 Hz, 1H), 7.56 (t, *J* = 7.7 Hz, 1H); ^13^C-NMR (CDCl_3_) δ 24.4, 43.1, 71.9, 117.3, 117.8, 121.9, 134.5, 136.9, 157.1, 160.5; Anal. C_10_H_13_NO: Found C 73.69, H 8.15, N 8.49%; Calcd C 73.59, H 8.03, N 8.58%; [α]_D_^25^ −14.7° (c 0.95, CHCl_3_) for 84% ee; Daicel CHIRALCEL OD, UV Detector 254 nm, hexane/*i*-PrOH = 30:1, flow rate 0.5 mL/min. t_R_ = 12.0 min (*R*), 13.8 min (*S*).

*1-(p-Dimethylaminophenyl)-3-buten-1-ol* (**5d**) [7]. IR (neat) ν 3435, 2802, 1614, 1522, 1348, 1162, 1052, 915, 819 cm^−1^; ^1^H-NMR (CDCl_3_) δ 1.89 (d, *J* = 2.6 Hz, 1H), 2.45-2.58 (m, 2H), 2.95 (s, 6H), 4.65 (ddd, *J* = 7.1, 6.2, 2.6 Hz, 1H), 5.11 (dm, *J* = 10.2 Hz, 1H), 5.15 (dm, *J* = 17.2 Hz, 1H), 5.82 (dddd, *J* = 17.2, 10.2, 7.3, 6.9 Hz, 1H), 6.72 (d, *J* = 8.8 Hz, 2H), 7.24 (d, *J* = 8.8 Hz, 2H); ^13^C-NMR (CDCl_3_) δ 40.7, 73.4, 112.6, 117.9, 126.9, 131.9, 135.1, 150.3; [α]_D_^24^ −51.8° (c 0.35, CHCl_3_) for 84% ee; Daicel CHIRALCEL OD, UV Detector 254 nm, hexane/i-PrOH = 9:1, flow rate 0.5 mL/min. t_R_ = 15.9 min (minor), 18.5 min (major). 

*(E)-1-(p-Dimethylaminophenyl)-1,5-hexadien-3-ol* (**5e**). IR (neat) ν 3674, 1730, 1610, 1522, 1437, 1352, 968, 806 cm^−1^; ^1^H-NMR (CDCl_3_) δ 1.75 (d, *J* = 3.7 Hz, 1H), 2.36 (ddddd, *J* = 14.0, 7.4, 6.9, 1.1, 1.0 Hz, 1H), 2.43 (ddddd, *J* = 14.0, 6.8, 5.4, 1.4, 1.2 Hz, 1H), 2.95 (s, 6H), 4.31 (dddd, *J* = 7.2, 6.9, 5.4, 3.7 Hz, 1H), 5.14 (dddd, *J* = 10.2, 2.1, 1.2, 1.0 Hz, 1H), 5.17 (dddd, *J* = 17.1, 2.1, 1.4, 1.1 Hz, 1H), 5.86 (dddd, *J* = 17.1, 10.2, 7.4, 6.8 Hz, 1H), 6.03 (dd, *J* = 15.8, 6.9 Hz, 1H), 6.50 (d, *J* = 15.8 Hz, 1H), 6.67 (d, *J* = 8.9 Hz, 2H), 7.27 (d, *J* = 8.9 Hz, 2H); ^13^C-NMR (CDCl_3_) δ 40.6, 42.2, 72.4, 112.5, 118.2, 125.1, 127.2, 127.5, 130.8, 134.5, 150.3; Anal. C_14_H_19_NO: Found C 77.30, H 8.89, N 6.44%; Calcd C 77.38, H 8.81, N 6.45%; [α]_D_^25^ −21.5° (c 1.08, CHCl_3_) for 88% ee; Daicel CHIRALCEL OD, UV Detector 254 nm, hexane/*i*-PrOH = 9:1, flow rate 0.5 mL/min. *t*_R_ = 20.0 min (minor), 21.1 min (major). 

*1-Acetoxy-1-(2-pyridyl)-3-methyl-3-butene* (**6b**-**Ac**). IR (neat) ν 3076, 2933, 1742, 1651, 1591, 1472, 1236, 894 cm^−1^; ^1^H-NMR (CDCl_3_) δ 1.77 (s, 3H), 2.11 (s, 3H), 2.65 (d, *J* = 6.8 Hz, 2H), 4.72 (bs, 1H), 4.79 (bs, 1H), 5.98 (t, *J* = 6.8 Hz, 1H), 7.20 (dd, *J* = 7.7, 4.8 Hz, 1H), 7.31 (d, *J* = 7.7 Hz, 1H), 7.67 (td, *J* = 7.7, 1.7 Hz, 1H), 8.60 (dd, *J* = 4.8, 1.7 Hz, 1H); ^13^C-NMR (CDCl_3_) δ 21.1, 22.6, 74.7, 113.7, 121.2, 122.8, 136.7, 141.2, 149.5, 159.3, 170.4; Anal. C_12_H_15_NO_2_: Found C 70.29, H 7.29, N 6.76%; Calcd C 70.22, H 7.37, N 6.82%; [α]_D_^26^ −37.5° (c 1.08, CHCl_3_) for 51% ee; Daicel CHIRALCEL OD, UV Detector 254 nm, hexane/*i*-PrOH = 50:1, flow rate 0.5 mL/min. *t*_R_ = 17.8 min (*R*), 21.0 min (*S*).

*1-(6-Methyl-2-pyridyl)-3-methyl-3-buten-1-ol* (**6c**). IR (neat) ν 3399, 2924, 1645, 1591, 1458, 1156, 889 cm^−1^; ^1^H-NMR (CDCl_3_) δ 1.82 (bs, 3H), 2.37 (dd, *J* = 14.0, 8.8 Hz, 1H), 2.52 (dd, *J* = 14.0, 4.4 Hz, 1H), 2.55 (s, 3H), 4.14 (d, *J* = 4.4 Hz, 1H), 4.81 (bs, 1H), 4.85 (dt, *J* = 8.8, 4.4 Hz, 1H), 4.89 (bs, 1H), 7.04 (d, *J* = 7.8 Hz, 1H), 7.08 (d, *J* = 7.8 Hz, 1H), 7.56 (t, *J* = 7.8 Hz, 1H); ^13^C- NMR (CDCl_3_) δ 22.7, 24.4, 47.4, 70.9, 113.6, 117.3, 121.9, 136.9, 142.5, 157.2, 161.1; Anal. C_11_H_15_NO: Found C 74.58, H 8.53, N 7.80%; Calcd C 74.54, H 8.53, N 7.90%; [α]_D_^20^ −29.1° (c 1.37, CHCl_3_) for 45% ee; Daicel CHIRALCEL OD, UV Detector 254 nm, hexane/*i*-PrOH = 30:1, flow rate 0.5 mL/min. *t*_R_ = 12.5 min (*R*), 13.5 min (*S*).

*1-(p-Dimethylaminophenyl)-3-methyl-3-buten-1-ol* (**6d**). White solid. mp. 28–30 °C; IR (neat) ν 3251, 2886, 2800, 1524, 1442, 1350, 1162, 1054, 816 cm^−1^; ^1^H-NMR (CDCl_3_) δ 1.79 (bs, 3H), 1.98 (bs, 1H), 2.39 (dd, *J* = 14.1, 4.4 Hz, 1H), 2.48 (dd, *J* = 14.1, 9.2 Hz, 1H), 2.94 (s, 6H), 4.74 (ddd, *J* = 9.2, 4.4, 1.5 Hz, 1H), 4.85 (bs, 1H), 4.90 (bs, 1H), 6.73 (d, *J* = 8.8 Hz, 2H), 7.26 (d, *J* = 8.8 Hz, 2H); ^13^C-NMR (CDCl_3_) δ 22.5, 40.8, 48.0, 71.4, 112.6, 113.7, 126.9, 132.0, 142.9; Anal. C_13_H_19_NO: Found C 76.07, H 9.29, N 6.77%; Calcd C 76.06, H 9.33, N 6.82%; [α]_D_^19^ −55.7° (c 1.41, CHCl_3_) for 90% ee; Daicel CHIRALCEL OD, UV Detector 254 nm, hexane/*i*-PrOH = 9:1, flow rate 0.5 mL/min. *t*_R_ = 15.0 min (*R*), 17.4 min (*S*).

*(E)-1-(p-Dimethylaminophenyl)-3-methyl-1,5-hexadien-3-ol* (**6e**). IR (neat) ν 3631, 3397, 2926, 2801, 1611, 1447, 1167, 965, 804 cm^−1^; ^1^H-NMR (CDCl_3_) δ 1.79 (d, *J* = 2.8 Hz, 1H), 1.80 (bs, 3H), 2.34 (d, *J* = 6.6 Hz, 2H), 2.96 (s, 6H), 4.40 (tdd, *J* = 6.6, 6.6, 2.8 Hz, 1H), 4.85 (bs, 1H), 4.90 (bs, 1H), 6.03 (dd, *J* = 15.7, 6.6 Hz, 1H), 6.54 (d, *J* = 15.7 Hz, 1H),6.68 (d, *J* = 8.8 Hz, 2H), 7.28 (d, *J* = 8.8 Hz, 2H); ^13^C-NMR (CDCl_3_) δ 22.7, 40.6, 46.5, 70.6, 112.5, 113.6, 125.3, 127.6, 130.0, 130.5, 142.4, 150.3; Anal. C_15_H_21_NO: Found C 76.07, H 9.29, N 6.77%; Calcd C 76.06, H 9.33, N 6.82%; [α]_D_^20^ −30.2° (c 1.66, CHCl_3_) for 84% ee; Daicel CHIRALCEL OD, UV Detector 254 nm, hexane/*i*-PrOH = 9:1, flow rate 0.5 mL/min. *t*_R_ = 20.0 min (*R*), 21.1 min (*S*).

### 3.4. Synthesis and X-ray Analysis of (i-Pr-Phebox)RhCl_2_ (**κ-3a**)

*(i-Pr-Phebox)RhCl_2 _*(κ-**3a**). To a stirred solution of *i*-Pr-**1** (200 mg, 0.41 mmol) in dichloromethane (5 mL) was added **3a** (39 μL, 0.41 mmol) at ambient temperature. After it was stirred for 2 h, the mixture was concentrated under reduced pressure. Purification of the residue by silica gel chromatography (dichloromethane/ether = 1:1) gave (*i*-Pr-Phebox)RhCl_2_(κ-**3a**) in 84% yield (200 mg). Orange solid. mp. 203-205 °C (decomp); IR (KBr) ν 2958, 1711, 1620, 1485, 1394, 1214, 963, 739 cm^−1^; ^1^H-NMR (CDCl_3_) δ 0.64 (d, *J* = 6.8 Hz, 6H), 0.73 (d, *J* = 6.8 Hz, 6H), 1.37 (dsept, *J* = 2.8, 6.8 Hz, 2H), 4.04 (ddd, *J* = 10.0, 6.4, 2.8 Hz, 2H), 4.62 (dd, *J* = 8.8, 6.4 Hz, 2H), 4.74 (dd, *J* = 10.0, 8.8 Hz, 2H), 7.30 (t, *J* = 7.6 Hz, 1H), 7.65 (d, *J* = 7.6 Hz, 2H), 7.79 (dd, *J* = 7.6, 5.6 Hz, 1H), 8.49 (dd, *J* = 7.6, 1.6 Hz, 1H), 10.07 (dd, *J* = 5.6, 1.6 Hz, 1H), 10.27 (s, 1H), 10.30 (d, *J* = 1.6 Hz, 1H); ^13^C-NMR (CDCl_3_) δ 15.1, 19.2, 29.4, 66.9, 71.1, 123.3, 125.2, 128.1, 131.6, 132.6, 136.6, 155.1, 157.2, 172.4 (*J*_Rh-C_ = 3.4 Hz), 185.9 (*J*_Rh-C_ = 19.7 Hz), 189.8; Anal. C_24_H_28_Cl_2_N_3_O_3_Rh: Found C 49.62, H 4.86, N 7.22%; Calcd C 49.67, H 4.86, N 7.24%. X-ray-quality crystals of (*i*-Pr-Phebox)RhCl_2_(κ-**3a**) was obtained from benzene-ether-hexane at room temperature and mounted in glass capillary. Diffraction experiments were performed on a Rigaku AFC-7R four-circle diffractometer equipped with graphite-monochromated Mo K〈 radiation; ﾤ = 0.71069 Å. The lattice parameters and an orientation matrix were obtained and refined from 25 machine-centered reflections with 29.82 < 2╰ < 29.97°. Intensity data were collected using a ﾡ-2╰ scan technique, and three standard reflections were recorded every 150 reflections. The data were corrected for Lorentz and polarization effects. The structure was solved by direct methods [27] and expanded using Fourier techniques [28]. The non-hydrogen atoms were refined anisotropically. Hydrogen atoms were included but not refined. The final cycle of full-matrix least-squares refinement was based on 5306 observed reflections (*I* > 3∫(*I*)) and 598 variable parameters. Neutral atom scattering factors were taken from Cromer and Waber [29]. All calculations were performed using the teXsan crystallographic software package [30]. Final refinement details are collected in Table 5 and the numbering scheme employed is shown in Figure 2, which was drawn with ORTEP at 30% probability ellipsoid. Crystallographic data (excluding structure factors) for the structures reported in this paper have been deposited with the Cambridge Crystallographic Data Centre as supplementary publication no. CCDC-826794. Copies of the data can be obtained free of charge on application to CCDC, 12 Union Road, Cambridge CB21EZ, UK (fax: (+44)1223-336-033; Email: deposit@ccdc.cam.ac.uk).

**Table 5:** Crystallographic data and structure refinement for (*i*-Pr-Phebox)RhCl_2_(κ-**3a**).

Empirical Formula	C_48_H_58_N_6_O_7_Cl_4_Rh_2_	Temperature	23.0 °C	
Formula Weight	1178.65	Scan type	*ω* -2 *θ*	
Crystal Dimensions	0.15 × 0.5 × 0.5 mm	Scan Width		94
				3 tan *θ* deg
Crystal System	monoclinic	2*θ*_max_	55.0 deg	
Lattice Type	C-centered	No. of Reflection	Total: 6787	
Lattice Parameters: *a*	18.307(4) Å	measured		
*b*	14.886(5) Å	No. of Unique data	6581 (*R*_int_ = 0.018)	
*c*	21.056(4) Å	Structure Solution	Direct methods	
*β*	106.55(2) deg	Refinement	Full-matrix	
Volume	5500(2) Å^3^		least squares	
Space Group	*C*2 (#5)	No. of Observations	5306 (I>3*σ*(I))	
*Z* value	4	No. of Variables	598	
*D*_calcd_	1.423 g/cm^3^	Reflection/Parameter	8.87	
*F*(000)	2408.00	Ratio		
*μ*(Mo Kα)	8.44 cm^−1^	Residuals: *R*; *R*_w_	0.058; 0.077	
*λ*	0.71069 Å			

## 4. Conclusions

In this paper, we have described the catalytic enantioselective addition of allyl- and methallyltributyltin reagents to amino aldehydes catalyzed with air-stable and water tolerant chiral Phebox-Rh(III) aqua complexes. The reactions proceed under mild conditions to afford the corresponding homoallylic alcohols with modest to good enantioselectivity (up to 94% ee), and these aqua complexes can be recovered from the reaction media by column chromatography. We have clarified that the chiral (Phebox)RhX_2_ fragments (X = Cl, Br), generated by releasing water molecule from (Phebox)RhX_2_(H_2_O), capture amino aldehydes at the carbonyl oxygen and the reaction proceeded *via* a Lewis acid mechanism.
